# Left ventricular kinetic energy as a marker of mechanical dyssynchrony in failing hearts with LBBB: a 4D flow CMR study

**DOI:** 10.1186/1532-429X-18-S1-O91

**Published:** 2016-01-27

**Authors:** Jakub Zajac, Jonatan Eriksson, Petter Dyverfeldt, Urban Alehagen, Tino Ebbers, Ann Bolger, Carl Johan Carlhall

**Affiliations:** 1 Division of Cardiovascular Medicine, Department of Medical and Health Sciences, Linköping University, Linköping, Sweden; 2 Department of Cardiology, Department of Medical and Health Sciences, Linköping University, Linköping, Sweden; 3 Department of Medicine, University of California San Francisco, San Francisco, CA USA

## Background

Left bundle branch block (LBBB) leads to dyssynchronous left ventricular (LV) contraction and relaxation which may contribute to LV dysfunction and ultimately heart failure. LBBB-related mechanical dyssynchrony often responds to cardiac resynchronization therapy (CRT). However, this therapy is expensive and the number of non-responders remains significant. Reliable functional markers of dyssynchronous LV pumping that can predict response to CRT have proved elusive.

Almost all studies of LV mechanical dyssynchrony focus on wall motion properties rather than aspects of intraventricular flow. 4D flow CMR specific measures have recently emerged as markers of LV function in failing hearts. Reduced volume and kinetic energy (KE) of the portion of LV inflow which passes directly to outflow (*Direct Flow*) has been demonstrated in failing LVs compared to normal LVs. In this study we hypothesized that the volume and KE of *Direct Flow* would be further reduced in myopathic LVs with LBBB compared to similarly dysfunctional and remodeled LVs without LBBB.

## Methods

22 heart failure patients were enrolled; 11 patients with LBBB and 11 patients without LBBB matched according to LV ejection fraction (EF), LV end-diastolic volume (EDV) index, heart rate, age and gender. In both groups, etiology of heart failure was dilated cardiomyopathy in 7, and ischemic cardiomyopathy in 4. 4D flow CMR and morphological images were acquired on a 3T Philips Ingenia. A previously validated method was used for flow analysis (Eriksson et al., JCMR 2010): Pathlines were emitted from the ED LV blood volume and traced forward and backward to the time of ES, thus encompassing one cardiac cycle. The traced ED blood volume was separated into 4 functional flow components. The kinetic energy (KE) of each flow component was computed over the cardiac cycle from the volume represented by each trace, its velocity and the density of blood.

## Results

**Figure 1:**
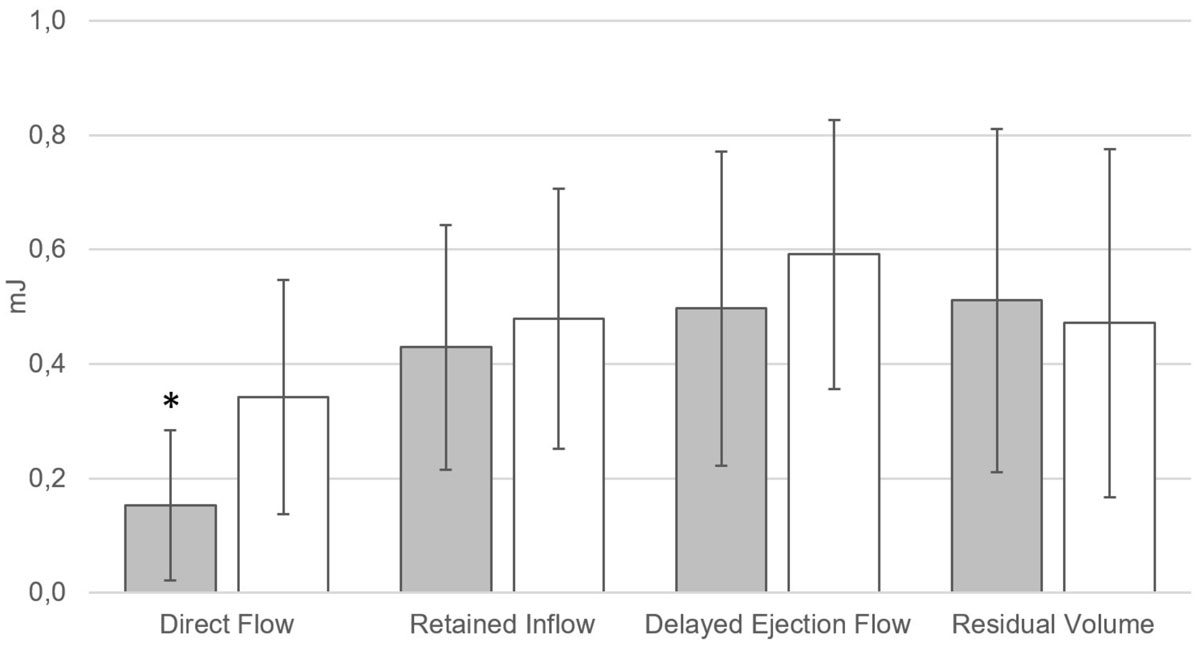
**Kinetic energy at end-diastole (mJ) for the 4 flow components in LBBB patients (grey) and matched patients without LBBB (white)**. mJ, millijoule. *P = 0.018 vs *Direct Flow* in patients without LBBB.

There was no intergroup difference in LVEF, LVEDV-index, heart rate and age, whereas the QRS duration was longer (P < 0.001) in patients with LBBB (Table 1). The volume of the *Direct Flow* component was not significantly different between the groups (P = 0.17), but the KE at end diastole was lower among patients with LBBB (P = 0.018, Figure 1). When normalized to the *Direct Flow* volume, *Direct Flow* KE at ED was lower in patients with LBBB compared to matched patients without LBBB (P = 0.007, Table 1).

**Table 1:** Clinical characteristics and 4D flow CMR data of patients with and without LBBB

	With LBBB (n = 11)	Without LBBB (n = 11)	P-value
Age (years)	61 ± 14	57 ± 15	0.599
Gender (female:male)	2:9	2:9	-
Heart rate (bpm)	71 ± 13	68 ± 10	0.551
LVEDV-index (ml/m^2^)	129 ± 48	115 ± 32	0.434
LVEF (%)	34 ± 9	36 ± 9	0.588
QRS duration (ms)	160 ± 20	104 ± 10	< 0.001
**Volume (ml)**			
Direct flow	18.7 ± 10.5	25.7 ± 12.6	0.174
Retained inflow	54.5 ± 16.4	48.4 ± 16.6	0.393
Delayed ejection flow	48.7 ± 15.6	45.8 ± 11.6	0.631
Residual volume	119.7 ± 73.2	98.2 ± 63.3	0.471
**KE/volume at ED (mJ/ml)**			
Direct flow	0.007 ± 0.003	0.013 ± 0.005	0.007
Retained inflow	0.008 ± 0.002	0.010 ± 0.004	0.079
Delayed ejection flow	0.010 ± 0.005	0.013 ± 0.005	0.128
Residual volume	0.004 ± 0.001	0.005 ± 0.002	0.563

Mean ± SD. ED, end diastole; EDV, end-diastolic volume; EF, ejection fraction; KE, kinetic energy; LV, left ventricle

## Conclusions

4D flow patterns and energetics in myopathic LVs with and without LBBB demonstrate reduced end-diastolic KE of *Direct Flow* in patients with LBBB compared to matched patients with normal conduction. This may reflect incremental impairment of diastolic function and less efficient ensuing ejection related to dyssynchrony in these failing ventricles. These intriguing preliminary findings suggest that 4D flow specific measures reflect LV mechanical dyssynchrony in heart failure patients, and could be investigated as predictors of response to CRT.

